# Systemizing and Transforming Preterm Oral Feeding Through Innovative Algorithms

**DOI:** 10.3390/children12040462

**Published:** 2025-04-03

**Authors:** Rena Rosenthal, Jean Chow, Erin Sundseth Ross, Rudaina Banihani, Natalie Antonacci, Karli Gavendo, Elizabeth Asztalos

**Affiliations:** 1 Department of Newborn and Developmental Paediatrics, Sunnybrook Health Sciences Center, 2075 Bayview Avenue, Toronto, ON M4N 3M5, Canada; jean.chow@sunnybrook.ca (J.C.); rudaina.banihani@sunnybrook.ca (R.B.); natalie.antonacci@sunnybrook.ca (N.A.); karli.gavendo@sunnybrook.ca (K.G.); elizabeth.asztalos@sunnybrook.ca (E.A.); 2 Department of Pediatrics, University of Colorado School of Medicine, University of Colorado Anschutz Medical Campus, Denver, CO 80045, USA; eross@feedingfundamentals.com; 3 Department of Paediatrics, University of Toronto, 555 University Avenue, Toronto, ON M5G 1X8, Canada; 4 Occupational Science and Occupational Therapy, Temerty Faculty of Medicine, University of Toronto, Toronto, ON M5G 1V7, Canada

**Keywords:** oral feeding, cue-based feeding, preterm infants, outcomes

## Abstract

**Background:** Establishing safe and efficient oral feeds for preterm infants is one of the last milestones to be achieved prior to discharge home. However, this process commonly elicits stress and anxiety in both care providers, such as nurses and the entire healthcare team in the Neonatal Intensive Care Unit (NICU), as well as parents. These feelings of uncertainty are exacerbated by the non-linear progression of oral feeding development and the absence of a systematized approach to initiate and advance feedings. **Methods:** In this 48-bed tertiary perinatal centre, staff surveys and a needs assessment showed dissatisfaction and increasing stress and anxiety due to the inconsistencies in initiating and advancing oral feeds. This paper describes the formation of a multidisciplinary feeding committee which reviewed various oral feeding training materials and the ultimate creation of two innovative oral feeding algorithms and their corresponding education materials. **Results:** The Sunnybrook Feeding Committee has developed two evidence-based algorithms, one for initiating oral feeds and another for monitoring progress with objective decision-making points during common oral feeding challenges. To complement and support these algorithms, educational materials and a comprehensive documentation process were also created. These resources included detailed instructions, visual aids, and step-by-step guides to help staff understand and apply the algorithms effectively. Additionally, the educational materials aimed to standardize training and ensure consistency across the NICU, further promoting a systematic approach to preterm oral feeding. Implementation of these algorithms also aimed to provide evidence-based, expert-guided guidelines for assessing readiness, initiating feeds, monitoring progress, and making necessary adjustments. **Conclusions:** This structured approach lays the foundation for a unit-wide language and systematic process for oral feeding. The next steps in this quality improvement project involve educating and piloting the implementation of the developed oral feeding algorithms, gathering staff feedback, and refining the tools accordingly. The goal is to enhance overall care quality, reduce stress for both care providers and parents, and ensure the best possible start for vulnerable preterm infants, ultimately supporting a smooth and successful transition to home.

## 1. Introduction

Preterm infants are born with immature physiological systems, and their journey through the Neonatal Intensive Care Unit (NICU) is marked by the gradual maturation of these systems [1,2]. Among the various milestones they must achieve during their NICU stay, the development of oral feeding skills, encompassing the intricate coordination of the suck–swallow–breathe sequence, tends to be one of the last to mature [1,3]. Achieving these skills is often a prerequisite for their eventual discharge home [4,5,6]. The acquisition and refining of these vital oral feeding skills hinge upon many influential factors. These factors encompass the infant’s gestational age, feeding experiences, environmental factors, the level of parental involvement, as well as the presence of significant neonatal morbidities, which can include chronic lung disease, a need for surgical interventions, and the need for prolonged ventilation for any reason [7,8,9].

Oral feeding is a multifaceted developmental process influenced by many variables, including maturation, physiological factors, and environmental dynamics [10,11,12]. This inherent complexity contributes significantly to the challenges NICU staff encounter when introducing, advancing, and establishing safe oral feeds for preterm infants [13]. Feeding practices within many NICUs often lack uniformity and have traditionally relied on a trial-and-error approach, nursing experience or established customs that frequently lack a foundation in evidence-based guidelines [14,15]. Many NICUs do not rely on written guidelines for initiating oral feeds but tend to determine the initiation of oral feeds based on the infant’s corrected gestational age and other developmental considerations [16]. Consequently, these challenges can result in prolonged gavage feeding, extended hospitalization stays, heightened stress among staff, and increased anxiety among parents [17]. Moreover, they may lead to feeding-related difficulties, including avoidance and aversion, further complicating the infant’s journey towards successful oral feeding.

Oral feeding readiness involves two key oral feeding opportunities: First, recognizing when an infant who has primarily been receiving gavage feeds shows signs of readiness to start oral feeding, and second, assessing their ability to actively participate in each specific oral feeding session [15,16,17]. It is important to note that an infant may not show signs of oral feeding readiness at every feeding opportunity. This distinction emphasizes the transition from volume-based gavage feeding to cue-based feeding, moving from scheduled feeds to modified ad-lib or on-demand feeding. This approach has been shown to decrease the time to full oral feeds, increase parental involvement in the feeding process, and reduce the length of hospital stay [18,19]. A comprehensive approach and expert training for care providers is essential to support preterm infants throughout their feeding journey, ensuring appropriate initiation of oral feeds and advancing each feeding session based on the infant’s cues.

Assessing oral feeding readiness is crucial in helping care providers determine the appropriate timing for initiating and advancing oral feeding [20,21]. Various assessment tools exist for this purpose, including the Neonatal Oral-Motor Assessment Scale (NOMAS), the Parents-Child Interaction Feeding Scale (formally known as the Nursing Child Assessment Satellite Training Scale, NCAST), and the Infant Driven Feeding Scales. However, these tools must be adapted for use in preterm settings, even though they may not have been specifically designed or validated for preterm infants. Additionally, the tool must be elegant enough to encompass many facets of readiness, including physiologic, motor, and arousal state behaviours. Most of these tools are designed primarily for bottle feeders and may not be applicable to both breast and bottle feeding. They also may require extensive care provider training, rendering them impractical for clinical care settings [22,23]. Given the complexities and the need for ongoing, individualized support during an oral feeding experience, it is also essential to guide care providers on what actions to take during feeding [12].

Sunnybrook Health Sciences Centre (SHSC) is one of three high-risk regional neonatal centres servicing the Central East region of Ontario, Canada. We provide integrated family-centred care for infants requiring Level III neonatal care. Our team, comprised of physicians, nurse practitioners (NPs), respiratory therapists (RTs), nurses (RNs), dietitians (RDs), lactation consultants (LCs), physiotherapists (PTs), and occupational therapists (OTs), supports multidisciplinary family-centred care approach to all aspects of care, including feeding. Each of these disciplines, along with the parents and family, is actively involved in creating and adjusting the feeding plan.

Over the years, it was becoming increasingly clear that our unit would benefit from a more structured and streamlined approach to oral feeding, one that was not dependent on the skills or experience of the individual feeder but rather based on the infant’s unique needs (considering medical comorbidities as well as feeding development, behaviours, and skills). The ongoing turnover of bedside staff, including the retirement of experienced nurses and the influx of newly hired staff with limited experience in feeding fragile preterm infants, further highlighted the need for a systematic approach to oral feeding.

Recognizing these limitations, we identified the need for a more structured, consistent and evidence-based approach to oral feeding in this unique population of fragile feeders. Fragile feeders, typically preterm or medically compromised infants, require careful, ongoing assessment and individualized support due to their physiologic instability, immature oromotor skills, and behavioural disorganization [24,25,26].

This developed oral feeding approach would not only leverage care provider expertise but also focus on understanding and responding to the infant’s cues. Our primary goal was to develop an approach that ensures a safe and developmentally appropriate oral feeding experience for these vulnerable infants and their feeder (care provider or parent) while promoting a systemized feeding process.

## 2. Materials and Methods

This quality improvement (QI) initiative was conducted at Sunnybrook Health Sciences Centre, a tertiary perinatal care unit in Toronto, Ontario, Canada.

### 2.1. Baseline and Needs Assessment

In our NICU, oral feeding has always been deeply rooted in the traditional practices—”how things were always done”—rather than being guided by research and evidence-based practices.

The initial phase of this initiative involved identifying factors that had historically impeded the effective management of oral feeding for preterm infants in the NICU setting.

**Figure 1:**
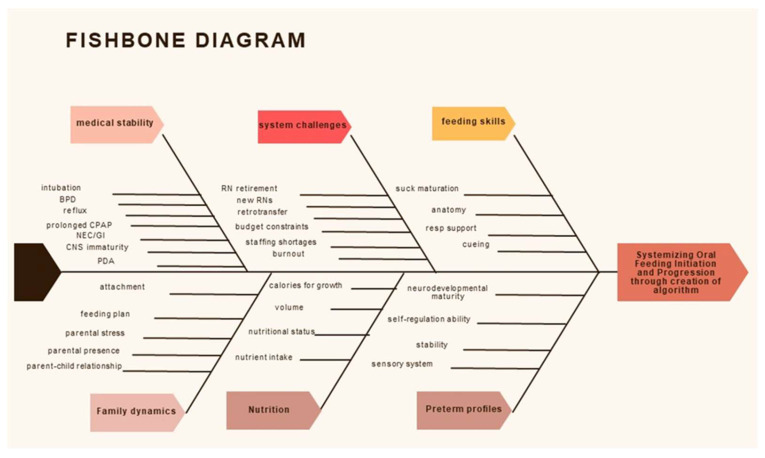
Fishbone Diagram: Visual representation of these factors, serving as a foundational step in addressing the challenges and streamlining the process to enhance the care of preterm infants during their oral feeding development.

Figure 1 provides a visual representation of these factors, serving as a foundational step in addressing the challenges and streamlining the process to enhance the care of preterm infants during their oral feeding development.

### 2.2. Staff Surveys and Gap Identification

**Figure 2:**
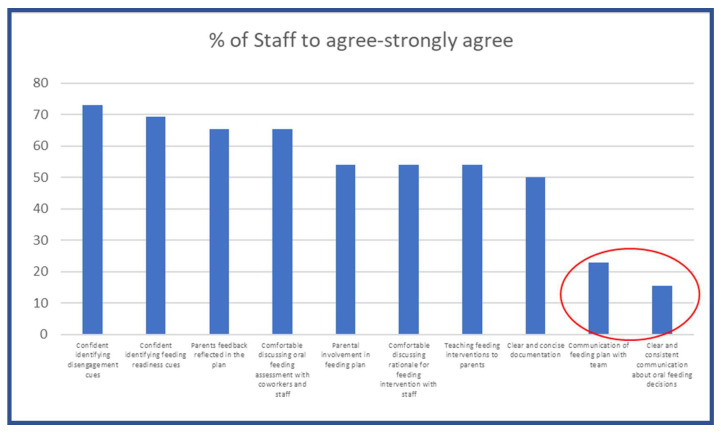
Modified Pareto Chart demonstrating the poor communication of the oral feeding plan with the team, as well as clear and consistent communication about oral feeding decisions.

Staff satisfaction surveys completed in 2020, during the COVID-19 pandemic, revealed that stress and anxiety increased due to the inconsistencies in initiating and advancing oral feeds. Additionally, staff expressed dissatisfaction with various aspects of the process, including the development of feeding plans, communication within the healthcare team, responsiveness to feedback from parents and staff, as well as the documentation of these crucial elements. As illustrated in Figure 2, staff did not feel the oral feeding plan was well communicated with the rest of the team or that there was consistent communication surrounding oral feeding decisions.

### 2.3. Formation of Feeding Committee and Review of Educational Materials

In response to these surveys, the need to create a consistent and evidence-based oral feeding approach was identified, and the Sunnybrook Feeding Committee was established. This was a multidisciplinary team of healthcare professionals, including physicians, nurses, nurse practitioners, occupational therapists, and dietitians. The committee’s mission was to develop a systematic approach, applicable to both breast and bottle feeding, with a common language for assessing and communicating information related to the initiation and progression of oral feeding. The goal was to empower both the multidisciplinary NICU staff, as well as parents, to become proficient in understanding their infant’s feeding cues and ensuring a safe and positive oral feeding experience.

The Feeding Committee’s initial focus was to create a systematic approach to oral feeding for the NICU staff, especially the bedside nurses. Training bedside nurses would not only improve the infant’s care during their NICU stay but also enable the nurses to aid parents in understanding and implementing this method effectively, further enhancing the infant’s feeding journey and overall well-being [27]. Furthermore, this unit-wide consistency in initiating and advancing oral feeds would also help streamline the activation and allocation of next steps and additional resources during the infant’s NICU stay, such as referrals for oral feeding assessments, to ensure timely support and tailored oral feeding plans.

Various published feeding approaches, as outlined earlier, were reviewed and considered by the Feeding Committee as a basis for understanding and educating staff on the various oral feeding cues used to assess oral feeding readiness and advancement. Starting this initiative during a global COVID-19 pandemic required the use of virtual educational content due to the strict isolation requirements in hospitals. The decision was made to utilize the Supporting Oral Feeding in Fragile Infants (SOFFI^®^) education modules, as all the teaching modules were available online. Additionally, SOFFI^®^ was evidence-based, with a study on its implementation in a children’s hospital setting with both extremely premature and ill infants [27]. In that study, a core group received initial training, while the rest of the staff were given a much shorter version of the training focused on using the SOFFI^®^ algorithms. The feeding committee began with an initial 25 staff members completing 13 h of SOFFI^®^ education, with a goal of training all staff over several years. The creator of SOFFI^®^ (Consultant) worked with members of the Feeding Committee to individualize the education modules and develop materials to better suit our hospital and patient population. Specifically, the SOFFI^®^ algorithms were used as part of the original training but were adapted to focus more on the healthy, developing preterm infant [27,28].

The Feeding Committee and Consultant adapted the original SOFFI^®^ algorithms to develop specific oral feeding algorithms suited to our hospital. Given that all documentation is recorded electronically, we felt it was acceptable to split and recombine the original algorithms in ways that would be easily incorporated into the computerized charting. The new algorithms continue to streamline and standardize the oral feeding experience for care providers by providing a universal language to facilitate effective communication and discussion of all feeding plans and progression.

## 3. Results

### 3.1. Adapted Education Module

A foundational education module based on the core principles of the SOFFI^®^ training was created and deemed mandatory for all staff through the Sunnybrook Online Learning Management System (LMS). This provided the basic principles and language so all staff could start becoming familiar with the universal language of preterm infants and their oral feeding cues. This training utilized videos of fragile feeders within our unit to demonstrate the various engagement and disengagement cues, as well as having a mandatory passing quiz at the end to prove completion and assess learning. Using this LMS, we were able to familiarize staff with the basics of the SOFFI^®^ oral feeding cues before they completed the formal training.

### 3.2. Sunnybrook Feeding Algorithms

The Sunnybrook Feeding Committee and Consultant adapted and developed two main algorithms: an Oral Feeding Readiness Algorithm and an Oral Feeding Challenges Algorithm. These algorithms were exclusively intended for NICU staff use, with the goal of creating later versions with language suitable for educating parents.

**Figure 3:**
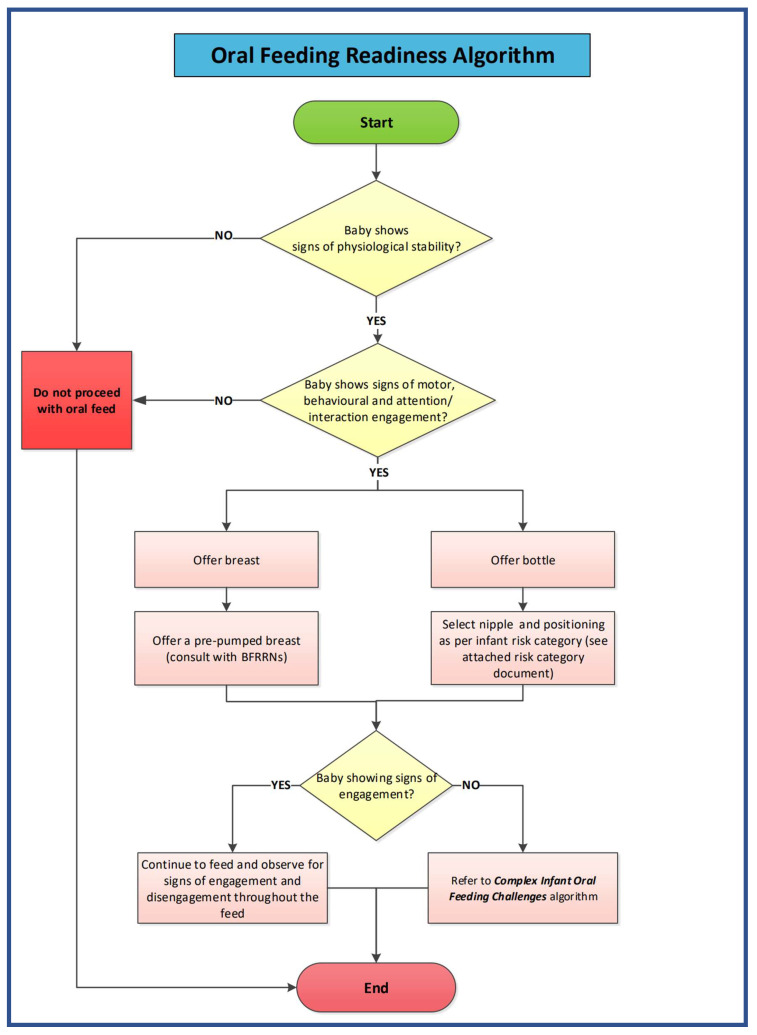
Sunnybrook NICU Oral Feeding Readiness Algorithm. © 2024 Sunnybrook Health Sciences Centre/Sunnybrook Research Institute and Erin S Ross. All rights reserved. These algorithms have been collaboratively developed by Sunnybrook Health Sciences Centre/Sunnybrook Research Institute and Erin S Ross, incorporating the foundational principles of the SOFFI^®^ method. The SOFFI^®^ principles and product are protected under a separate copyright and trademark held by Dr. Erin S Ross, all rights reserved. Sunnybrook Health Sciences Centre/Sunnybrook Research Institute acknowledge the contributions of Dr. Erin S Ross, the creator of SOFFI^®^, in the foundational principles and any excerpts of the SOFFI^®^ materials utilized in these algorithms. Any unauthorized use, in whole or in part, reproduction or distribution of these algorithms without explicit permission from Sunnybrook Health Sciences Centre/Sunnybrook Research Institute and Dr. Erin S. Ross is strictly prohibited. For permissions or inquiries, please initially contact Sunnybrook Health Sciences Centre/Sunnybrook Research Institute.

**Figure 4:**
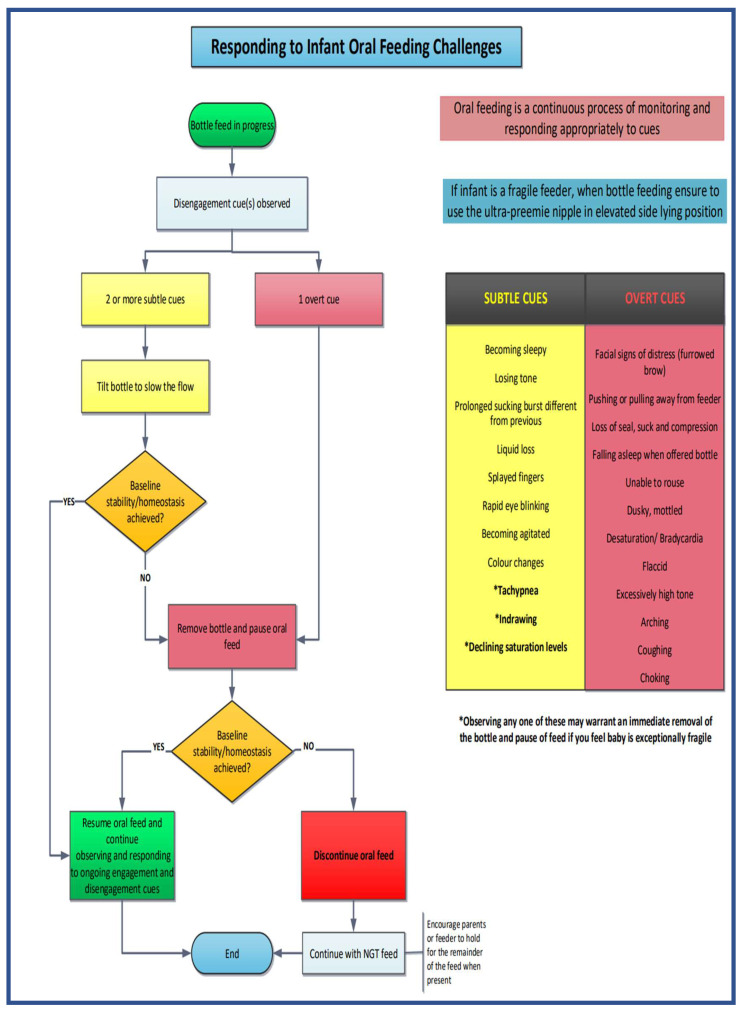
Sunnybrook NICU Oral Feeding Challenges Algorithm. © 2024 Sunnybrook Health Sciences Centre/Sunnybrook Research Institute and Erin S Ross. All rights reserved. These algorithms have been collaboratively developed by Sunnybrook Health Sciences Centre/Sunnybrook Research Institute and Erin S Ross, incorporating the foundational principles of the SOFFI^®^ method. The SOFFI^®^ principles and product are protected under a separate copyright and trademark held by Dr. Erin S Ross, all rights reserved. Sunnybrook Health Sciences Centre/Sunnybrook Research Institute acknowledge the contributions of Dr. Erin S Ross, the creator of SOFFI^®^, in the foundational principles and any excerpts of the SOFFI^®^ materials utilized in these algorithms. Any unauthorized use, in whole or in part, reproduction or distribution of these algorithms without explicit permission from Sunnybrook Health Sciences Centre/Sunnybrook Research Institute and Dr. Erin S. Ross is strictly prohibited. For permissions or inquiries, please initially contact Sunnybrook Health Sciences Centre/Sunnybrook Research Institute.

The Oral Feeding Readiness Algorithm (Figure 3) assesses oral feeding readiness both for the first time and at the start of each oral feeding opportunity. It has been designed to apply to both breast and bottle feeds and is suitable for preterm and term infants. The Oral Feeding Challenges Algorithm (Figure 4) is tailored specifically for fragile preterm feeders (born at less than 26 weeks’ gestation) and focuses exclusively on bottle feeds.

Crucially, both algorithms emphasized that oral feeding is an ongoing process that requires continuous monitoring and the care provider’s close attention to recognize and respond to the infant’s feeding cues, whether they are signs of engagement or disengagement [24,29].

#### 3.2.1. Decision to Initiate an Oral Feed (Oral Feeding Readiness Algorithm) (Figure 3)

Physiological stability formed the algorithm’s cornerstone and served as the fundamental basis for initiating oral feeds since the emphasis aligns with the core of the synactive theory [30]. If an infant is not physiologically stable before the initiation of feeds, they may struggle to self-regulate and maintain homeostasis, especially when confronted with additional physiologic demands of oral feeding [9,29,31].

When an infant is showing signs of physiological instability, such as mottled or dusky skin colour, oxygen desaturations, bradycardic episodes, and/or breathing rates that deviate from the infant’s baseline, the care provider must refrain from initiation of that oral feed. Instead, the care provider should opt for gavage feeding as the safer alternative. This decision is viewed positively as it reflects and confirms the care provider’s ability to recognize and appropriately respond to physiologic disengagement cues, ensuring the infant’s safety and well-being [32].

The algorithm initiates at the “Start” point by evaluating the infant’s baseline fundamental physiologic stability. The assessment encompasses various factors, including evaluating the infant’s skin colour (ranging from pink to dusky), respiratory rate, oxygen saturation level, and heart rate.

When an infant demonstrates physiologic stability, the subsequent step involves assessing other vital systems, including motor, behavioural, and attention/interaction. This comprehensive evaluation ensures a cohesive picture of an infant who displays engagement cues across all these realms. An infant with behaviours signifying their readiness to participate in the feeding process at that particular feeding session will likely be more successful than an infant not showing readiness behaviours [33,34].

Readiness cues encompass various indicators, such as the infant independently rousing with little assistance, moving into flexion, displaying smooth movements, arousing with non-nutritive sucking (NNS), and showing interest or cueing indicating a desire to feed. The care provider will become trained to recognize and understand these diverse cues and confidently make a “YES” or “NO” decision when assessing for proceeding with that particular oral feed to offer either breast or bottle feeding based on parental preference and the established feeding plan.

If this is the first attempt at the breast, a pre-pumped breast will be offered, and the breastfeeding resource nurse (BFRN) will be consulted.

If bottle feeding is chosen, the care provider will adhere to the medical order specifying the appropriate nipple and bottle system based on the infant’s specific needs, ensuring a tailored approach to feeding.

Suppose the infant shows more disengagement cues for that particular feed, a “NO” decision in the algorithm. In that case, the care provider will not proceed with that oral feed and will offer a gavage feed instead. The care provider can then perform the same assessment the next time the infant shows engagement cues and interest in oral feeding.

The care provider will continually assess the monitoring of the infant’s cues throughout the oral feed. If the infant continues to show engagement cues in all systems—motor, behavioural, and attention/interaction—the feed can continue to proceed.

#### 3.2.2. Decisions During an Oral Feed (Oral Feeding Challenges Algorithm) (Figure 4)

This algorithm was specifically designed for infants classified as fragile feeders, including those born under 26 weeks of gestation, who are bottle-feeding using the slowest flow nipple, the ultra-preemie, and positioned safely for oral feeds in an elevated side-lying position.

An infant enters this algorithm when, during a bottle feed, the care provider observes signs of disengagement, as described in the training modules [32]. The care provider will then classify the disengagement cue on the continuum, ranging from subtle to more overt, to determine the appropriate course of action.

Within the algorithm, disengagement cues have been categorized into two groups: subtle cues (shown on the algorithm in yellow) and more overt cues (shown on the algorithm in red). These colour-coded ranges, from yellow to red, depict the continuum of disengagement cues from the subtle cues, which are more benign cues that may allow for intervention on behalf of the care provider, to the more overt and potent cues that necessitate termination of that oral feeding opportunity.

##### Subtle Disengagement Cues

Subtle disengagement cues include various signs that indicate the infant may be experiencing challenges during the oral feed. These cues include the infant becoming drowsy, sleepy, losing muscle tone, having prolonged sucking bursts leading to a challenge maintaining oxygen levels, experiencing liquid loss from their mouth, displaying splayed fingers, rapid eye blinking, agitation, and changes in skin colour. These behaviors often lead to tachypnea, indrawing, and declining oxygen saturation levels. The progression of these subtle disengagement behaviors illustrates an increasing stress level and difficulty for the infant to cope with the demands of the oral feed.

When the infant shows two or more of these identified subtle disengagement cues, the care provider can attempt a corrective action. This involves tilting the bottle to slow the feed flow, which can help bring the infant back to their baseline stability. If this intervention results in the infant returning to a state of baseline stability, indicated by a “YES” response, the oral feed can continue. The care provider must remain vigilant throughout the feed, continually observing and responding to the infant’s engagement and disengagement cues.

However, if, despite the intervention of tilting the bottle to slow the feed, the infant is unable to return to baseline stability, marked by a “NO” response, the bottle must be removed, and the oral feed should be paused. Suppose the act of pausing the oral feed allows the infant to regain baseline stability, indicated by a “YES” response. In that case, the care provider can then resume the oral feed, maintaining continuous observation and responsiveness to the infant’s cues throughout the remainder of the feeding session.

##### Overt Disengagement Cues

Overt disengagement cues represent more pronounced and potent signs of infant distress during oral feeding. These cues encompass various indicators such as furrowed brow (or other facial expressions of distress), pulling away from the feeder, loss of seal, cessation of sucking or nipple compression, falling asleep or being unable to rouse during a feed, displaying dusky or mottled skin, experiencing bradycardias or desaturations, exhibiting flaccidity or excessively high muscle tone, arching, and coughing or choking. Like the subtle cues, the progression of these overt cues along the continuum indicates an increasing level of stress and the infant’s difficulty in maintaining stability during the oral feed.

If the disengagement cue observed at any point throughout the feed is classified as overt, the care provider must promptly remove the bottle and allow the infant a break to pause to help resume baseline stability. If the momentary pause results in the infant achieving baseline stability, indicated by a “YES” response, the oral feed can be resumed, and the care provider should continue to observe and respond to the infant’s engagement and disengagement cues throughout the oral feed.

However, if, despite the pause in oral feeding by removing the bottle, the infant cannot regain baseline stability, denoted by a “NO” response, the oral feed must be discontinued. In such cases, the remaining feed volume should be administered via the nasogastric tube (NGT). Care providers are encouraged to hold the infant during the NGT feed, which can help foster positive associations between feeding and comfort [12,19,35,36].

As long as the infant remains physiologically stable and displays signs of engagement, oral feeding can proceed, with the care provider maintaining an ongoing assessment of the infant’s cues to ensure a safe and positive feeding experience.

##### Decision to End an Oral Feed

At any point during the oral feeding, the infant may begin to exhibit disengagement cues. This can occur either because the infant has consumed an adequate volume for that particular feed or because the infant is starting to lose stability in one of its physiological systems. The care provider will constantly monitor these cues, which will serve as the basis for discontinuing the oral feed and marking the end of the algorithm for that particular feeding session.

However, when the infant starts to show signs of physiological stability along with engagement cues in their other systems (motor, behavioral, and attention/interaction), the care provider will once again assess the infant’s readiness for feeding and initiate the algorithm anew for the next feeding opportunity, ensuring that the infant’s well-being and progress are continuously monitored and supported.

### 3.3. Documentation

The feeding committee decided to integrate the algorithms into the hospital’s electronic medical record and utilize these algorithms as the foundation for documentation as well as a means of improving communication between team members in the creation and assessment of feeding plans. The care provider will use the online drop-down options integrated into the electronic charting system to accurately record their “YES” or “NO” decision-making points at each algorithm stage. Additionally, care providers will comment on any subtle or overt disengagement cues observed during the oral feed.

This documentation approach not only ensures a systematic and consistent record of the infant’s feeding progress but also facilitates improved communication among the healthcare team. Addressing this aspect of communication was identified as a priority for enhancement through staff surveys, and tracking the accuracy and frequency of oral feeding documentation will contribute significantly to addressing this need. It will foster a more cohesive and well-informed approach to the infant’s feeding plan and the decisions made throughout the feeding process, including consistent swallowing and feeding assessment referral criteria.

## 4. Discussion

Establishing safe and efficient oral feeding in the NICU is a critical milestone for preterm infants and often a prerequisite for their eventual discharge home. This responsibility typically falls upon the care provider, whether it be the medical/professional staff or the parent, to ensure that the infant is acquiring and reinforcing the necessary skills for safe oral feeding.

Feeding experiences must be seen as conversations between the feeder and the infant. These decision-making points in these algorithms are driven by the various engagement and disengagement cues the infant is displaying before and during each feed. Initiation of an oral feed is dependent on stable physiological cues. Physiological disengagement cues are critical signs that the infant will not effectively tolerate the added stressor of an oral feed and could potentially deteriorate during such an oral feeding attempt. Continually offering oral feeds, despite these signs of physiological instability, can lead the infant to develop disorganized/compensatory (e.g., liquid loss, non-nutritive suck) eating patterns as a coping mechanism to maintain homeostasis throughout the feeding process.

Moreover, in alignment with the Neuronal Grouping Selection Theory and Classical Conditioning, each negative feeding experience can lead the infant to develop aversive associations with oral feeding [11,37]. Over time, as these negative associations accumulate, the infant’s overall perception of oral feeding can become increasingly negative, potentially compounding feeding challenges and aversions. This underscores the significance of recognizing and responding appropriately to engagement and disengagement cues to promote an infant’s positive and successful oral feeding experience [24,32].

The algorithms provide an organized, evidence-based and systematic approach for initiating and progress oral feedings, with the infant’s behaviour guiding and individualizing the interventions that may be used [38] They serve a dual purpose: firstly, to assess oral feeding readiness comprehensively; and secondly, to provide a structured framework for interventions that can support feeding challenges for infants in the NICU throughout the feeding process, one that does not depend on the skills or experience of the feeder but is entirely driven by the infants and their feeding cues. This approach reduces the reliance on subjective decisions made by care providers, which previously had a substantial impact on oral feeding practices in the NICU setting.

Importantly, these algorithms address a crucial need for improved communication among care providers, ultimately striving to ensure a positive and successful oral feeding experience for both infants and their care providers. The algorithms provide a structured way to help the feeder truly focus on what the infant is saying, which decreases the variability during feedings unrelated to the infant’s needs.

## 5. Next Steps

Following the introduction and implementation of these algorithms, the next phase of this project is to educate staff and assess their competency using these algorithms to ensure an understanding of oral feeding cues and appropriate decision-making at each stage of the algorithms. This will involve care providers being observed by two other team members who are deemed “experts” with the algorithms to assess their understanding of the various steps, communication within the team, as well as adherence to and consistent documentation of the oral feeding experience. These assessments and testing will be an important step in validating that these adapted algorithms continue to focus the feeder on the infant’s behaviours.

Staff deemed competent with understanding and executing the algorithms will then be eligible to train parents on understanding and responding to their infant’s various oral feeding cues.

Finally, the last phase of this QI initiative will be to conduct a thorough review after 12 months of implementing these algorithms to assess care providers’ compliance and determine whether oral feeding, in general, becomes a more positive experience for the infant and their care provider. The anticipated findings from this evaluation will ideally serve as a foundation for enhancing the overall oral feeding setting and experience for parents caring for their preterm infants in the NICU, both leading up to discharge home and during the crucial first few weeks post-discharge.

## 6. Conclusions

This QI initiative represented a broader effort to transform oral feeding practices in the NICU, establishing a foundation for consistent knowledge transfer and scalable feeding support. The development and integration of these algorithms aim to provide a reliable and evidence-based approach that aligns with infant cues, facilitating a smooth transition from NICU to home. Moving forward, the next phase will focus on educating NICU staff, implementing and refining the algorithms within our NICU, adapting them for parental use, and developing a comprehensive training program for families to support safe, consistent feeding practices post-discharge.

In summary, this structured approach will provide NICU staff with tools for confident, informed feeding care, thereby reducing stress and enhancing oral feeding outcomes for preterm infants. The ongoing work underscores the critical role of knowledge-based protocols in clinical practice, advancing sustainable knowledge transfer across caregivers and contributing to the long-term developmental success of preterm infants.

## Data Availability

The original contributions presented in this study are included in the article. Further inquiries can be directed to the corresponding author.
